# Molecular docking analysis of estrogen receptor binding phytocomponents identified from the ethyl acetate extract of *Salicornia herbacea (L)*

**DOI:** 10.6026/97320630018273

**Published:** 2022-03-31

**Authors:** Venkatesan Rama, Bharathi Muruganantham, Kanakasabapathi Devaki, Sridhar Muthusami

**Affiliations:** 1 Department of Biochemistry, Karpagam Academy of Higher Education, Tamil Nadu, Coimbatore 641 021, India; 2 Department of Biochemistry, Vivekanandha College of Arts & Sciences for Women (Autonomous), Tiruchengode - 637205, India

**Keywords:** *S. herbacea*, HPTLC, GC-MS, Phytoconsitituents, ER, di-n-octyl phthalate

## Abstract

It is of interest to evaluate the secondary metabolites using high performance thin layer chromatography (HPTLC) finger printing and Gas chromatography-Mass spectroscopy (GC-MS) in *S. herbacea*extract. The powdered plant material extracted using different solvents were used for the qualitative analysis of alkaloids, flavonoids, terpenoids and saponins followed by HPTLC finger printing and GC-MS analysis. The components identified in the GC-MS were docked with estrogen receptor (ER) to identify the binding specificity of isolated compounds. The ethyl acetate extract of *S. herbacea*showed the presence of high number of secondary metabolites when compared to other solvent system. The qualitative analysis of the plant material also showed the presence of carbohydrates, protein, amino acid, phenol, flavonoids, terpenoids, glycosides, saponins and steroids. The HPTLC finger printing analysis revealed the existence of alkaloid, flavonoid, terpenoid and saponin compounds and GC-MS. GC-MS was performed to identify the phytocomponents constituents in the extract. 8 phytocompounds were identified to analyse binding with ER. The binding affinity score (-6.8 kcal/mol) and interacting ER residues (28) the phyto compound di-n-octyl phthalate showed best docking score with ER α than the standard drugs lasofoxifene, and 4-hydroxytamoxifen. The binding affinity and number of interacting ER residues was -6.9 kcal/mol; 10 and -6.2; 11, respectively. The results identified the presence of ER antagonist in *S. herbacea*and warrants further investigation to explore for treating ER regulated diseases.

## Background:

Plants are genuinely utilized as a tool for the extraction and isolation of active compounds that resulted in the discovery of new drugs with high therapeutic values [1]. Medicinal plants can act in a symbiotic manner within the human body and present exclusive -therapeutic properties with minimal or no desired side effects [2]. Different phytoconstituents of herbal products are documented to be beneficial for the treatment of diseases caused by free radicals. It also protects the body from tissue injury [3]. Phytochemicals comprises the secondary compounds of plant where as the chlorophyll, proteins and common sugars are included in the primary constituents. Phytochemicals include terpenoids, alkaloids, glycosides and phenolic compounds [4]. The latter exhibits the various important pharmacological activities in oxidative stress, inflammation, diabetes, asthma, hepatitis, cancer and gastro [5,6]. HPTLC and High performance liquid chromatography (HPLC), emerge as the efficient tools for phyto-chemical evaluation, and enable the analysis of several samples simultaneously [7]. It reduces both time and cost analysis and ensures the reusability of identified spots for quantification by densitometry in a specific track, called as a fingerprint [8-10]. S. herbace, commonly known as pickle weeds glasswort, belongs to Amaranthaceae family [11]. The name *S. herbacea*has been originated from the Latin word meaning 'salt'. Studies have been reported that some species, of *S. herbacea*shows tolerance towards salinity as high as 3 % NaCl [12]. This plant is found at the edges of wetlands, marshes, sea shores, and mudflats [13 - check with author]. ER has provided us with a powerful prognostic and therapeutic marker as well as a promising target for anti-estrogen treatment for hormone-dependent breast cancer [14,15] for drug discovery [16-17]. Therefore, it is of interest to evaluate the secondary metabolites using high performance thin layer chromatography (HPTLC) finger printing and Gas chromatography-Mass spectroscopy (GC-MS) in *S. herbacea*extract.

## Materials and Methods:

### Plant collection and authentication:

*S. herbacea*was collected from Ennore area, Chennai. The species was authenticated by Professor Jayaraman, Plant Anatomy Research Center, West Thambaram, and Chennai and assigned a (Voucher number: PARC/2014/2028).

### Extraction of plant material:

The dried sample was coarsely powdered and stored in a sterile container. A total 50 g of dried plant powder was subjected to successive solvent extraction using five different solvents such as petroleum ether, chloroform, ethyl acetate, ethanol and water in the ratio of 1:5 using Soxhlet apparatus for 24-48h. Obtained extract was evaporated for dryness by using a rotary vacuum evaporator at 40-50°C and stored at 0-4°C in an air tight until further use.

### Phytochemical screening of phytoconstituents:

The phytochemical analysis of the plant extracts was carried out to find out the phytochemical constituents such as carbohydrates, proteins, amino acids, tannins, glycosides, alkaloids, terpenoids and flavonoids by using standard methods [18,19]. The yield of the various solvents was also recorded.

### HPTLC finger printing analysis of ethyl acetate extract of *S. herbacea*plant:

10 µl each of the plant samples was dissolved in 50 µl ethyl acetate and diluted. This diluted sample was centrifuged at 3000 rpm for 5 minutes. 2 µl of sample were loaded as 5 mm band rpm length in the 3x10 silica gel 60F254 TLC Plate using Hamilton syringe and CAMAG LINOMAT 5 Instrument. The sample loaded plate was kept in TLC twin through developing chamber with mobile phase, ethyl acetate- methanol water (5:3:1), n-Hexane -Ethyl acetate (7.2:2.9), Chloroform-Glacial acetic acid-Methanol-Water (6.4:3.2:1.2:0.8) Alkaloid, flavonoid, terpenoid, saponin respectively. The developed plates were dried and documented using CAMAG REPROSTAR 3 at visible light, 254 nm and 366 nm the plates were fixed in a scanner stage and scanned at 254 nm. The peak Table, display and densitogram were noted using win (ATS 1.3.4 version for derivation, respective spray reagents were used for detecting bands and the plates were documented in visible light and 366 nm [20].

### Analysis of GC-MS:

The Clarus 680 GC become used inside the evaluation that's hired to a fused silica column, filled with Elite-5MS (5% biphenyl 95% dimethylpolysiloxane, 30 m x 0.25 mm identity x 250µm df) and the components had been separated using Helium as service fuel at a regular flow of 1 ml/min. at some stage in the chromatographic run the injector temperature changed into set at 260°C. The 1µl of extract pattern injected into the tool and the oven temperature become as follows: 60°C (2 min); observed by using 300°C on the charge of 10°C min-1; and 300°C, where it became held for six minutes. The mass detector situations including switch line temperature 240°C; ion source temperature 24°C; and ionization mode electron effect at 70 eV, an experiment time 0.2 sec and test c language of 0.1 sec. The fragments are from 40 to 600 Da. The spectrums of the components have been as compared with the database of the spectrum of the recognized additives saved in the GC-MS NIST (2008) library 27 [21].

### Target preparation and Ligand Library:

The crystallographic structure of ER (PDB ID: 6VJD) alpha ligand-binding domain was retrieved from the protein data bank (PDB) with the ligand lasofoxifene. The downloaded ER structure was edited to remove the ligand lasofoxifene and water molecules using the Discovery Studio Visualizer v19.1.0.18287 (www.accelerys.com) and again saved in PDB format. The major phytoconstituents present in *S. herbacea*were retrieved in SDF format from the PubChem database. The obtained phytocompounds were then converted into PDB file format using OPEN BABEL software [22]. Also, the native ligand lasofoxifene and 4-hydroxytamoxifen were selected as standard drugs and docked with ER to compare the effect of *S. herbacea* phytocompounds.

### Molecular Docking:

After preparing the phytocompounds as ligands and ER as a target, PyRx was implied with the Autodock Vina option using the new scoring function [23]. It analyzes the docking orientations and interactions between the ligands and ER. The gird box properties were set as size x = 60.6778268703, size y = 72.6589010655 and size z = 76.405641152 for molecular docking. The significant interaction between the ligands and the receptors-binding site were acquired in 2D and 3D format by importing the docked results into the LigPlot+, PyMol and Discovery studio visualizer v19.1.0.18287 (www.accelerys.com).

### Evaluation of Ligands Drug likeness and Toxicity:

The screened ligands were evaluated for the drugability, physicochemical properties, toxicity, toxicity classes, and lethal dose using Molinspiration server (www.molinspiration.com/cgi-bin/properties). The drugability properties were analyzed based on the Molar Weights (MW), Total polar surface area (TPSA), lipophilicity (log P), Hydrogen Bond Acceptor (HBA), Hydrogen Bond Donor (HBD) to identify Lipinski's rule of the drug-like compounds. In addition, the Simplified Molecular Input Line Entry System (SMILES) were downloaded from the PubChem Database to calculate ADMET properties with toxicity class. The ADMET properties were calculated by implementing ADME Tlab 2.0 [24].

## Results and Discussion:

The phytochemical screening of the medicinal plants are important since it have commercial interest in both research institutes and pharmaceutical companies in manufacturing the novel drugs used for treatment of various diseases. The preliminary phytochemical screening of *S. herbacea*shows the presence of carbohydrates, proteins, amino acids, alkaloids, flavonoids, glycosides, saponins, steroids, phenol, tannins and terpenoids. The results of phytochemical analysis are tabulated in the Table 1(see PDF). Among the various extracts most of the phytochemicals are found in ethyl acetate extract of S. herbacea. The Table 2(see PDF) shows how the percentage yield of the different extracts of *S. herbacea*is. HPTLC is an effective analytical method. This method is visual, speedy and reasonable as it utilizes smaller quantities of solvents with minimum sample smooth up. Particularly, in a brief duration a huge number of samples are analyzed simultaneously [25]. HPTLC profile of ethyl acetate extract of *S. herbacea*was recorded in Tables 3, 4, 5, 6 and Figure 1-8(see PDF) for alkaloids, flavonoids, saponins and terpenoids respectively. The extracts had been run at the side of the standards consisting of colchicine, quercetin, lupeol and saponin respectively.

GC-MS evaluation led to the identity of wide variety of compounds from the GC fractions of the ethyl acetate extract of *S. herbacea*. These compounds were diagnosed through MS attached with GC. The compounds gift within the ethyl acetate extract of *S. herbacea*diagnosed via GC-MS evaluation as shown in Figure 9(see PDF). The active standards with their retention time (RT), molecular formula, molecular weight (MW) and concentration (%) within the ethyl acetate one extract of *S. herbacea*are presented in Table 7(see PDF). The prevailing compounds in the ethyl acetate extract were identified using library prediction as 2-PENTADECANONE, 6, 10, 14-TRIMETHYL23.68 % N-HEXADECANOIC ACID 23.68% OLEIC ACID 39.65%, 1-HEXYL-2-5.26%,16-HEPTADECENAL 4.67%, DI-N-OCTYL PHTHALATE 3.50%, BICYCLO [3.2.1] OCT-3-EN-2-ONE, 3,8-DIHYDROXY-1-METHOXY-7-(7-METHOXY-1,3-2.94%. The phytocompounds and their biological activities obtained through GC-MS study of *S. herbacea*.

According to the phyto-component of DI-N-OCTYL PHTHALATE has highly binding affinity to ER as evidenced through molecular docking analysis. The crystallographic structure of ER (PDB ID: 6VJD) α ligand-binding domain consisting of chain A, chain B, chain C and Chain D [26] were depicted in Figure 10(see PDF). The docked phytocompounds and target interacting residues with their binding affinity were tabulated in Table 8(see PDF). The assessment of crude extract is an imperative a part of accurate identification. HPTLC is useful as a phytochemical marker and more effective in the field of plant taxonomy and also for the identification of plant secondary metabolites [27]. HPTLC finger printing is proved to be a linear, unique, and correct technique for herbal identification. Such finger printing is useful in the quality control of herbal products and checking for the adulterants. Therefore, it may be beneficial for the assessment of various advertised pharmaceutical preparations. HPTLC profiles also show the occurrence of secondary metabolites of medicinal importance which support the traditional therapeutic uses of the plant species [28]. The qualitative analysis of ethyl acetate extracts of *S. herbacea*through HPTLC confirmed the presence of many secondary metabolites like alkaloids, flavonoids, saponins, and terpenoids (Figure 1-8 - see PDF)).

Four compounds with Rf values of 0.06, 0.84, 0.92, 0.94, are detected along with 3 unknown compounds (Table 3 - see PDF). In *S. herbacea*ethyl acetate extract in chromatogram (Figure 1 and 2 - see PDF), Orange, brown colored zone at visible mode is observed in the tracks which after a derivatization of brownish violet at 366 nm confirms the presence of alkaloid compound in the samples. The Table demonstrates that alkaloid numbered as 3 found to be maximum in its concentration. Alkaloids constitute one of the major groups of plant constituents. The mobile phase of used was ethyl acetate: methanol: water (10: 1.35: 1) for the alkaloid profiling. They represent one of the largest and most diverse families of the natural compound [29].

HPTLC of the ethyl acetate extract of *S. herbacea*plant Table 4(see PDF). Shows four peak areas, with four different Rf values. Among them, one peak shows the presence of flavonoid. With a Rf value of 0.85. The Figure 3 and 4(see PDF) exhibit the chromatogram and yellow and yellowish blue colored fluorescent zone at the 366 nm mode after derivatization confirms the presence of flavonoids in the sample Figure 3(see PDF). The mobile phase used is Ethyl acetate-Butanone-Formic acid-Water (5:3:1:1). Flavonoids are the most important natural phenolic and they possess a broad spectrum of chemical and biological activities including free radical scavenging properties. The flavonoids in plants have been reported to exert multiple biological effects including antioxidant, free radical scavenging abilities, anti-inflammatory, anti-carcinogenic effect, etc. [30].

The Table 5(see PDF) represents the saponin HPTLC profile of ethyl acetate extract of *S. herbacea*plant. In this profile, one standard is used, and one saponin is identified in the chromatogram of the extract. The Rf values in the reference standard and extract are found to be 0.07, 0.16, 0.22, 0.38, 0.92 and 041. Figure 5 and 6(see PDF) and peak 5 indicates the presence of saponin in *S. herbacea*plant. The band reveals the presence of saponin by its green, yellow, and blue colored zones at daylight mode after derivatization. Mobile phase consisting of chloroform: glacial acetic acid: methanol: water (6.4: 3.2: 1.2: 0.8) was used for profiling. Presence of saponins is important, since it exhibits a wide range of biological activities in controlling diabetes, cancer, bone health and stimulation of the immune system [31].

The chromatographic finger printing for terpenoids is well resolved at 366 nm after derivatization Figure 7 and 8(see PDF). The plates are sprayed with anisaldehyde sulphuric acid reagent followed by heating and visualized in day light which shows 10 prominent peaks in ethyl acetate extract. The 5, 6, 8, 9, & 10 peaks detect in the ethyl acetate extracts are identified as terpenoid and the best solvent system to scrutinize the above partition is n-hexane: ethyl acetate (7.2: 2.9). Most of the terpenoids are of plant origin; however, they are also synthesized by other organisms, such as bacteria and yeast as part of the primary or secondary metabolism. Terpenoids have been found to be useful in the prevention and therapy of several diseases, including oxidative stress, inflammation, diabetes, asthma, hepatitis, and cancer and gastro enteritis. A number of terpenoids exhibit cytotoxicity against an expansion of tumor cells and most cancers preventive in addition to anticancer efficacy in preclinical animal model [32].

GC-MS analysis caused the identification of wide variety of compounds from the GC fractions of the ethyl acetate extract of *S. herbacea*. These compounds have been diagnosed via MS connected with GC. The compounds present within the ethyl acetate extract of *S. herbacea*identified through GC-MS evaluation as shown in Figure 9(see PDF). The active concepts with their RT, molecular formula, MW and awareness (%) inside the ethyl acetate one extract of *S. herbacea*are supplied in Table 7(see PDF). The prevailing compounds in the ethyl acetate extract were 2-PENTADECANONE, 6,10,14-TRIMETHYL23.68 % N-HEXADECANOIC ACID 23.68% OLEIC ACID 39.65%, 2-PENTADECANONE, 6, 10, 14-TRIMETHYL23.68 % N-HEXADECANOIC ACID 23.68% OLEIC ACID 39.65%,1-HEXYL-2-5.26%,16-HEPTADECENAL 4.67%, DI-N-OCTYL PHTHALATE 3.50%, BICYCLO[3.2.1]OCT-3-EN-2-ONE, 3,8-DIHYDROXY-1-METHOXY-7-(7-METHOXY-1,3-2.94%. The phytocompounds and their biological activities obtained through GC-MS study of *S. herbacea* [33].

The 16-heptadecenal interacted with ALA430, ARG434, LEU509, ILE510, and HIS513 by alkyl interactions and is surrounded by the hydrophobic residues THR431 and GLN506. The predicted binding was noted as -3.3 kcal/mol with the RMSD 2.225 A. Di-n-octyl phthalate instituted the alkyl interactions withALA350, LEU354, TRP383, LEU384, LEU387, MET388, LEU391, PHE404, ILE424, LEU525, and PRO535 and surrounded by the hydrophobic residues MET343, THR347, LEU346, LEU349, ASP351, GLU353, ARG394, MET421, PHE425, LEU428, GLY521, HIS524 and VAL533. The binding affinity was predicted as -6.8 kcal/mol with the RMSD 1.681 A. Bicyclo[3.2.1]oct-3-en-2-one, 3,8-dihydroxy-1-methoxy-7-(7-methoxy-1,3-) significantly formed the hydrogen bond with SER512 and pi-sigma bond with ARG515. It also extends the alkyl interactions with HIS516, LEU511 and is surrounded by the hydrophobic residues ASN455 and ASN519 with binding affinity -5.5 and RMSD 2.366 A. The interacting residues with the selected standard drugs and other phytocompounds extracted from the *S. herbacea*were analyzed by LigPlot (Figure 11 - see PDF). The results explain that the compound di-n-octyl phthalate significantly inhibits ER than other phytocompunds from *S. herbacea*. The standard drug lasofoxifene-ER (Figure 11a - see PDF), 4-hydroxytamoxifen-ER (Figure 11e - see PDF), and di-n-octyl phthalate-ER (Figure 11i - see PDF) docked complex structure depicted in Figure 11(see PDF). Also, the nature of hydrogen bondonar and accpetor (Figure 11 b,f, j - see PDF), the 2D structure of the drug complex (Figure 11c, g, k - see PDF), and interacting residues by LigPlot (Figure 11d, h, l - see PDF) were delineated to identify the selected drug efficacy. The results evidently demonstrates that di-n-octyl phthalate shows potential inhibition effect against ER based on the binding affinity (-6.8 kcal/mol), RMSD (1.681 Å), and number of residues (No. 24) than the lasofoxifene (binding affinity = -6.9; RMSD = 4.741; No. = 10) and 4-hydroxytamoxifen (binding affinity = -6.2; RMSD = 1.941 ; No. = 11).

The native ligand lasofoxifene formed the hydrogen bond interaction with ARG434, alkyl interaction with ALA505, LEU509, ILE510 and hydrophobic interaction with ALA430, THR431, GLN502, GLN506 and SER512 residues with the binding affinity of -6.9 kcal/mol and RMSD 4.741. The standard drug 4-hydroxytamoxifen formed a carbon-hydrogen bond with SER512* and alkyl interaction with LEU508 and LEU509. It is also surrounded by the hydrophobic residues ILE451, ASN455, TYR459, LEU479, THR483, ALA505, LEU511, and ARG515 with the predicted binding affinity of -6.2 kcal/mol and RMSD 1.941 A. The phytocompound 2-Pentadecanone, 6,10,14-trimethyl interacted with HIS513* and HIS513 via carbon-hydrogen and pi-sigma bond. Also, it extends alkyl interaction with ARG434, LEU509, ILE510, HIS513 and is surrounded by the hydrophobic residues ALA430, THR431, SER512, and HIS516. The predicted binding affinity was noted as -4.2 kcal/mol with the RMSD 2.431 A. The N-Hexadecanoic acid exhibits alkyl interaction with ARG434, ILE510, HIS513 and is surrounded by hydrophobic residues such as ALA430 and THR431 LEU509, SER512, ARG515, HIS516, and ASN519. Also, the predicted binding affinity was observed as -3.9 kcal/mol with the RMSD 1.780 A. The 9,9-Dimethoxybicyclo[3.3.1]nona-2,4-dione interacted with LEU346*and GLY521* via carbon-hydrogen bond and extended alkyl interactions with ALA350 and LEU525. It is also surrounded by the hydrophobic residues MET343, THR347, TRP383, LEU384, LEU387, MET388, PHE404, MET421, and ILE424 with the predicted binding affinity of -5.8 kcal/mol and RMSD of 1.946. The oleic acid interacted with ALA430, ARG434, ILE510 and HIS513 through the alkyl interactions and was surrounded by the hydrophobic residues THR431, SER433, LEU509, SER512, and HIS516. Also, the predicted binding affinity was identified as -4.8 kcal/mol and the RMSD 2.02 A. The 1-Hexyl-2-nitrocyclohexane formed the carbon-hydrogen bond with GLY521* and establishes alkyl interaction with LEU346, ALA350, MET388, LEU391, PHE404, ILE424, and LEU428. It is also surrounded by hydrophobic residues such as MET343, LEU384, LEU387, MET421, PHE425, and LEU525 with the predicted binding affinity of -5.8 kcal/mol and RMSD 1.581 A.

In recent years, the complexity and risks of drug discovery and development procedures have grown significantly, resulting in greater expenditure on drug research [34,35]. The biopharmaceutical industry's productivity is declining due to poor ADMET (absorption, distribution, metabolism, excretion, and toxicity) qualities [36,37]. Oral administration is becoming the preferred method among patients due to its convenience and patient compliance [38,39]. In this heed, we have analyzed the ADMET properties for di-n-octyl phthalate and also for the selected standard drug lasofoxifene, and 4-hydroxytamoxifen to compare its efficacy (Table 9 - see PDF). For a new oral drug, bioavailability is one of the most desirable attributes. In contrast, assessing oral bioavailability is extremely difficult since bioavailability is a combined effect of numerous biological and physicochemical variables [40,41]. Here, we analyzed the bioavailability for the selected phytocompounds and then compared with the standard drugs. The bioavailability for the lasofoxifene, di-n-octyl phthalate was determined as F = <20% with the probability of 0.382 and 0.402, respectively. The 4-hydroxytamoxifen exhibits F = ≥20% with the probability of 0.589. As a result, the aforementioned event reminded us that human intestinal absorption (HIA) might serve as an alternate signal for oral bioavailability to some extent. As a result, it is also crucial in preclinical drug assessment [42-44]. The predicted HIA for the lasofoxifene, 4-hydroxytamoxifen and di-n-octyl phthalate is HIA = < 30%, with the probability of 0.716, 0.689, and 0.672. The human colon epithelial cancer cell line, caco-2, is used to model human intestinal absorption of drugs. The optimal value for the papp (caco-2 permeability) is >-5.15 or -4.70 or -4.80 cm/s. The estimated papp (caco-2 permeability) for the lasofoxifene, 4-hydroxytamoxifen,di-n-octyl phthalate is -5.141 cm/s, -5.002 cm/s, and -4.733 cm/s respectively. The predicted BBB (Blood–Brain Barrier) probability for lasofoxifene, di-n-octyl phthalate and 4-hydroxytamoxifen was 0.915, 0.7 and 0.995. It indicates all three drugs can penetrate the brain. Plasma protein binding (PPB) is a key criterion for a drug's effectiveness and safety to be explored during each drug-development program [45,46]. The plasma binding protein probability was identified as 89.32 %, 93.815 %, and 89.022 % for lasofoxifene, 4-hydroxytamoxifen, and di-n-octyl phthalate. The optimal value for the plasma binding protein is 90%. The higher the therapeutic index (TI), the safest the drug. If the TI is minimal (the difference in the two doses is extremely small), the medicine must be dosed cautiously [47,48]. The individual receiving the medicine should be continuously watched for any symptoms of drug toxicity. Therefore, the results suggested that all three drugs were safe for patients consuming. The CYP1A2 (Cytochromes P450) enzyme is responsible for the biotransformation of 8.9% of medicines that undergo hepatic metabolism.

CYP facilitates the metabolism of over half of all marketed medicines, making it the most significant enzyme in drug metabolism. The analyzed lasofoxifene, 4-hydroxytamoxifen, di-n-octyl phthalate, and related CYP inhibition probability were predicted as 0.19, 0.088, and 0.051. It implies that the drug will be digested and eliminated, lowering the drug concentration in the blood and preventing toxicity. The half-life (t1/2) of a drug is the time necessary to reduce its concentration in the body by one-half via excretion and is important in deciding dose frequency. The predicted half-life for the lasofoxifene, 4-hydroxytamoxifen, and di-n-octyl phthalate is 2.073 h, 2.243 h and 1.649 h, respectively, which explains the necessity of frequent doses in treatment. However, the phytocompound di-n-octyl phthalate extracted from the *S. herbacea*might be consumed as a decoction. Clearance rate (CL) is a proportionality factor that relates the concentration of drug measured in the body to the elimination rate. The identified clearance rate for lasofoxifene, 4-hydroxytamoxifen, and di-n-octyl phthalate was 1.826, 1.704, and 1.394 mL/min/kg. It described that the clearance rate was low might sustain in plasma for a long time. The hepatotoxicity (from hepatic toxicity) implies chemical-driven liver damage. The predicted hepatotoxic probability for the selected standard drugs lasofoxifene, 4-hydroxytamoxifen, and di-n-octyl phthalate was 0.868, 0.96, and 0.2. It distinctly describes that the standard drug will lead to high liver injury, and the phytocompound di-n-octyl phthalate is a hepatic-friendly drug.

## Conclusion:

Phyto constituents are identified by qualitative methods and the identified phyto constituents are ascertained using HPTLC. Ethyl acetate extract of *S. herbacea*plant is rich in terpenoids compounds with biological activities. The data based on the HPTLC finger print approach which can also be proposed as a quick and reliable analytic model for the pharmacognostic study of plant raw materials used in commercial products. Hence, the extracted phytocompounds from the plant *S. herbacea*using ethyl acetate was analyzed using molecular docking with ER. The phyto compound di-n-octyl phthalate extracted from the *S. herbacea*had the highest docking score towards ER,. Furthermore, the ADME/T characteristics of the di-n-octyl phthalate revealed that it might be deemed a potential drug-like chemical. The di-n-octyl phthalate is widely accessible, allowing for the earlier development of appropriate medications against estrogen driven breast cancer.
